# β-Lactam vs Non–β-Lactam Antimicrobial Prophylaxis and Surgical Site Infection

**DOI:** 10.1001/jamanetworkopen.2025.40809

**Published:** 2025-10-31

**Authors:** Selina Largiadèr, Delphine Berthod, Andreas Widmer, Nicolas Troillet, Holly Jackson, Christelle Perdrieu, Stephan Harbarth, Rami Sommerstein

**Affiliations:** 1 Faculty of Health Sciences and Medicine, University of Lucerne, Lucerne, Switzerland; 2 Service of Infectious Diseases, Central Institute, Valais Hospital, Sion, Switzerland; 3 Department of Infectious Diseases, University Hospital Basel, Basel, Switzerland; 4 Infection Control Division, Geneva University Hospitals and Faculty of Medicine, Geneva, Switzerland; 5 Department of Infectious Diseases, Bern University Hospital, University of Bern, Bern, Switzerland

## Abstract

**Importance:**

β-lactam–based surgical antimicrobial prophylaxis (SAP) is the standard for most surgical procedures. Alternatively, and mostly due to allergies, non–β-lactam–based prophylaxis is used. The association between the use of non–β-lactam SAP and an increased risk of surgical site infection (SSI) rate has not yet been conclusively described.

**Objective:**

To assess whether non–β-lactam vs β-lactam prophylaxis is associated with the occurrence of SSI.

**Design, Setting, and Participants:**

This cohort study was based on the Swissnoso SSI surveillance system of 175 hospitals, including adult patients who underwent a major surgical procedure with SAP administration within 120 minutes prior to incision and postdischarge follow-up from January 2009 to December 2020. Patients with wound contamination class IV were excluded. Data analysis was conducted from July to December 2024.

**Exposures:**

β-Lactam SAP vs non–β-lactam SAP.

**Main Outcomes and Measures:**

The main outcome was occurrence of SSI, according to Centers for Disease Control and Prevention definitions. Mixed-effects logistic regression models were used to adjust for institutional, patient, and perioperative confounding factors.

**Results:**

Of 538 976 surveilled patients, 348 885 (196 411 [56.3%] female; median [IQR] age, 63.2 [47.0-73.3] years) fulfilled eligibility criteria. β-Lactam SAP was administered in 342 936 patients (98.3%) and non–β-lactam SAP in 5949 patients (1.7%). SSI was diagnosed in 9507 patients (2.8%) exposed to β-lactam SAP vs 364 patients (6.1%) who received non–β-lactam SAP (*P* < .001). Non–β-lactam SAP was significantly associated with a higher SSI rate (adjusted odds ratio [aOR], 1.78; 95% CI, 1.59-1.99; *P* < .001) compared with β-lactam SAP. A higher SSI rate for non–β-lactam SAP was found across all procedure types. Secondary analyses found a higher risk of SSI for ciprofloxacin (aOR, 1.57; 95% CI, 1.33-1.87; *P* < .001), vancomycin (aOR, 1.38; 95% CI, 1.03-1.86; *P* = .04), and clindamycin (aOR, 2.12; 95% CI, 1.82-2.47; *P* < .001) compared with β-lactam SAP.

**Conclusions and Relevance:**

In this large cohort study, administration of non–β-lactam SAP was associated with 1.8-fold higher odds of SSI. These findings suggest that non–β-lactam SAP should be avoided whenever possible. A careful evaluation of patients with reported β-lactam allergy should be performed before administering a second-choice antibiotic.

## Key Points

**Question:**

Is the administration of non–β-lactam surgical antimicrobial prophylaxis (SAP) vs β-lactam SAP associated with increased surgical site infections (SSI)?

**Findings:**

In this cohort study of 348 885 patients who underwent 1 of 10 major surgical procedures, non–β-lactam SAP was associated with 1.8-fold higher odds of SSI.

**Meaning:**

These findings suggest that non–β-lactam SAP should be avoided whenever possible and for patients with reported β-lactam allergy delabeling should be enforced.

## Teaser

This cohort study compares risk of surgical site infection among patients treated with β-lactam vs non–β-lactam surgical antimicrobial prophylaxis.

## Introduction

Surgical site infections (SSIs) represent one of the most frequent complications following surgical procedures, contributing to patient morbidity and mortality, extended hospitalization periods, and substantial health care expenditures.^1,2,3,4,5^ To mitigate SSI risk, preoperative surgical antimicrobial prophylaxis (SAP) has become standard practice, with its optimal implementation (choice, dose, timing) requiring careful consideration of both antimicrobial efficacy against potential pathogens and associated adverse effects.^3,6,7,8^

β-Lactam antibiotics, in particular cefazolin and cefuroxime, are considered first-line agents for SAP for most surgical procedures due to their broad antimicrobial spectrum, bactericidal activity, half-life, and favorable safety profile.^7,8^ However, certain indications necessitate the use of alternative, non–β-lactam prophylactic agents with different mechanisms of action (such as protein synthesis inhibition), most notably in patients with self-reported or documented β-lactam allergies or when mandated by specific institutional guidelines.^7,8^

The comparative effectiveness of non–β-lactam vs β-lactam SAP in preventing SSIs remains incompletely characterized. While β-lactam antibiotics are preferentially recommended, studies suggest that non–β-lactam alternatives may be associated with elevated SSI rates. Current literature examining this association is limited by small sample sizes, insufficient adjustment for confounding factors, and inadequate longitudinal follow-up.^9,10,11^ Leveraging the comprehensive data available through our standardized national surveillance system, this study aimed to assess whether non–β-lactam SAP was associated with an increased risk of SSI compared with conventional β-lactam prophylaxis.

## Methods

### Study Design and Setting

This retrospective multicenter cohort study is based on prospectively collected and documented data from the Swissnoso SSI surveillance system, established in 2009, whose methods have been previously described and validated.^12,13,14,15,16^ Since this surveillance, mandated by the Swiss National Association for Quality Development in Hospitals and Clinics,^17^ is considered a quality-improvement project, no individual consent was required, per Swiss law, but all patients were informed about their inclusion and given the opportunity to opt out. The Bernese Cantonal Human Subjects Committee approved the analysis of risk factors for SSI using the Swissnoso database. Swissnoso publishes detailed summaries of the SSI rates and trends every year.^17^ The diagnosis of SSI was made independently from the surgeons: surgical patients were screened by infection control practitioners, and patients with suspected SSI were evaluated by a board-certified infectious disease specialist before being entered in the Swissnoso database. This study is reported following the Strengthening the Reporting of Observational Studies in Epidemiology (STROBE) reporting guideline.

We included all patients who received SAP from 175 participating health care institutions in Switzerland, which represent approximately 80% of all national hospitals performing surgical procedures, between January 2009 and December 2020. Participating institutions must cover at least 3 different surgical procedure types and have to include all patients who undergo these types of surgery. The collected surveillance data include baseline characteristics, surgical procedure, and outcomes at discharge as well as after discharge.^13,18^ Follow-up assessment occurred at 30 days after the procedure and at 1 year for implant procedures, per surveillance protocol. Through 5 contact attempts per patient, we achieved a postdischarge follow-up exceeding 91%.^13,18^

### Participants

**Figure 1.**
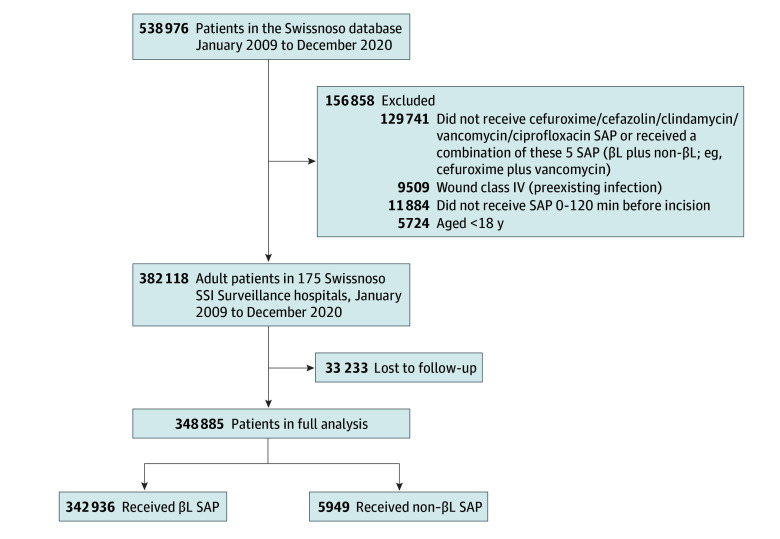
Participant Flowchart SAP indicates surgical antimicrobial prophylaxis; SSI, surgical site infection.

Patients participating in the surveillance system between 2009 and 2020 and meeting the following criteria were included: undergone 1 of the 10 most frequent surgical procedures (appendectomy, cardiac surgery, cesarean delivery, cholecystectomy, colorectal surgery, gastric bypass, hernia repair, hysterectomy, knee or hip arthroplasty, and spinal surgery [ie, laminectomy or spinal fusion]), age at least 18 years, and administration of either β-lactam SAP (cefuroxime or cefazolin ± metronidazole ± gentamicin) or non–β-lactam SAP (ciprofloxacin or vancomycin or clindamycin ± metronidazole ± gentamicin) within 120 minutes prior to incision. Patients were excluded if they had a high degree of wound contamination (class IV; ie, preexisting infection), their follow-up was incomplete, or they received both β-lactam and non–β-lactam antibiotics (excepted aminoglycosides and/or metronidazole) (Figure 1).

### Variables, Outcomes, and Data Sources

The primary outcome was SSI occurrence within 30 days (1 year for operations with implants), as defined by the Centers for Disease Control and Prevention.^19^ Swissnoso included a 1-year follow-up until 2023 to increase the sensitivity to detect low-grade infections, compared with 90 days in other surveillance systems. Secondary outcomes included SSI type (superficial incisional, deep incisional, or organ-space infection)^19^ and microbial etiology (when available). All suspected SSIs underwent validation by a dedicated physician (independent of the surgeon) before electronic documentation. Covariables included patient’s age; sex; American Society of Anesthesiologists (ASA) score, recorded according to the 1961 definitions^20^; wound contamination class (clean [class I], clean-contaminated [class II], or contaminated [class III]) according to Culver et al^21^; year of surgery; procedure type; hospital size (bed capacity); and procedure duration longer than the standard time (the 75th percentile of surgery time is referred to as the *procedure-specific T-time*).^18,22^

The exposure of interest was preincisional antimicrobial prophylaxis, administered within 120 minutes before incision, consisting of either β-lactams (cefuroxime or cefazolin) or non–β-lactams (ciprofloxacin, vancomycin, or clindamycin).^3^ These agents represent the most commonly used SAP at participating institutions, with specific selection dependent on institutional protocols.

For analysis, age was grouped into younger than 60 years and 60 years or older. To evaluate the associations of preoperative comorbidities, ASA scores were categorized into 2 groups: low (1-2) and high (3-5). Hospital sizes were grouped according to their number of beds: fewer than 200, 200 to 499 and 500 or more beds.

### Statistical Analysis

The χ^2^ and Wilcoxon test for categorical and continuous data, respectively, were used to investigate differences in baseline characteristics and SSI rates between SAP groups. SSI rates per type of surgical intervention were also calculated in both groups. SSI risks were estimated for non–β-lactam SAP compared with β-lactam SAP by fitting multilevel logistic regression models with clustering at the procedure level (random intercept), adjusted for all covariates. Missing data analysis included comparison of baseline characteristic of patients included and those lost to follow-up.

In addition, for each procedure type cluster, a 3:1 next-neighbor matching procedure between β-lactam and non–β-lactam groups based on propensity scores and with a maximum caliper of 0.1 was conducted to mitigate potential selection bias, account for disparities in baseline characteristics, and enhance the robustness of the comparative analysis. The propensity score model incorporated clinical and epidemiological important variables that may have confounded the results (age group category, ASA class, wound contamination class, and hospital bed size). In a next step, a logistic regression model with clustering at the procedure level (random intercept) was fitted to estimate SSI risks for the matched patients who received non–β-lactam SAP vs β-lactam SAP.

*P* values were 2-sided, and *P* < .05 was considered statistically significant. All analyses were performed in R software version 4.1.2 (R Project for Statistical Computing). Data analysis was conducted from July to December 2024.

## Results

Of 538 976 patients, 382 118 patients (70.9%) fulfilled eligibility criteria. Of these, 33 233 patients (8.7%) were lost to follow-up (Figure 1), leaving a total of 348 885 patients (196 411 [56.3%] female; median [IQR] age, 63.2 [47.0-73.3] years) included in the study. Characteristics of patients lost to follow-up and those included in the study are compared in eTable 1 in Supplement 1. The age distribution of both sexes can be seen in eFigure 1 in Supplement 1.

In this cohort, 342 936 patients (98.3%) received β-lactam SAP, while 5949 (1.7%) received non–β-lactam SAP (Figure 1). Comprehensive baseline patient demographics and procedural characteristics are presented for both groups in Table 1. The temporal distribution of SAP administration prior to surgical incision is illustrated in eFigure 2 in Supplement 1.

**Table 1.** Baseline and Procedural Characteristics of Patients by SAP Exposure Group

Characteristic	SAP exposure group, Patients, No. (%)		*P* value
	β-lactam (n = 342 936)	Non–β-lactam- (n = 5949)	
Age, median (IQR), y	63.18 (47.09-73.27)	61.78 (44.30-72.84)	<.001
Sex			
Female	192 347 (56.1)	4064 (68.3)	<.001
Male	150 589 (43.9)	1885 (56.1)	
ASA score			
1-2	245 251 (71.5)	3750 (63.0)	<.001
3-5	95 555 (27.9)	2175 (36.6)	
NA	2130 (0.6)	24 (0.4)	
Wound contamination class			
I (clean)	219 575 (64.0)	3057 (51.4)	<.001
II (clean-contaminated)	97 241 (28.4)	2142 (36.0)	
III (contaminated)	26 120 (7.6)	750 (12.6)	
Surgery exceeded standard time	58 651 (17.1)	1411 (23.7)	<.001
Year of procedure, median (IQR)	2015 (2013-2018)	2016 (2013-2018)	<.001
Hospital size, beds			
<200	188 469 (55.0)	2723 (45.8)	<.001
200-499	99423 (29.0)	1534 (25.8)	
≥500	55044 (16.1)	1692 (28.4)	
Endoscopic surgery			
No	230 069 (67.1)	3724 (62.6)	<.001
Yes	108 394 (31.6)	2098 (35.3)	
Beginning as endoscopy	4009 (1.2)	122 (2.1)	
Transvaginal procedure	357 (0.1)	4 (0.1)	
Transanal procedure	29 (<0.1)	1 (<0.1)	
NA	78 (<0.1)	0	
Implant	208 134 (60.7)	2749 (46.2)	<.001
Procedure type			
Appendectomy	6832 (2.0)	257 (4.3)	<.001
Cardiac surgery	24549 (7.2)	567 (9.5)	
Cesarean delivery	45 541 (13.3)	809 (13.6)	
Cholecystectomy	21 380 (6.2)	497 (8.4)	
Colorectal surgery	33 210 (9.7)	889 (14.9)	
Gastric bypass surgery	6871 (2.0)	208 (3.5)	
Hernia repair	31 528 (9.2)	500 (8.4)	
Hysterectomy	8005 (2.3)	178 (3.0)	
Knee and hip arthroplasty	148 514 (43.3)	1558 (26.2)	
Spinal surgery (laminectomy, spinal fusion)	16506 (4.8)	486 (8.2)	
Elective surgery	29 6545 (86.5)	4808 (80.8)	<.001
Choice of primary SAP			
Cefazolin	85 523 (24.9)		<.001
Cefuroxime	257 413 (75.1)		
Ciprofloxacin	0	1813 (30.5)	
Vancomycin	0	949 (16.0)	
Clindamycin	0	3187 (53.6)	
Addition of second SAP			
Gentamicin	6 (<0.1)	71 (1.2)	<.001
Metronidazole	40 894 (11.9)	1029 (17.3)	
NA	302 036 (88.1)	4849 (81.5)	
Addition of third SAP			
Gentamicin	1 (<0.1)	19 (0.3)	<.001
Metronidazole	79 (<0.1)	13 (0.2)	
NA	342 856 (100)	5917 (99.5)	

Abbreviations: ASA, American Society of Anesthesiologists; NA, not available; SAP, surgical antimicrobial prophylaxis.

Cefuroxime was administered to 257 413 patients (75.1%) and cefazolin to 85 523 patients (24.9%) who received β-lactam SAP. Clindamycin, ciprofloxacin, and vancomycin were administered in 3187 patients (53.6%), 1813 patients (30.5%), and 949 patients (16.0%) who received non–β-lactam SAP, respectively (Table 1). Adjunctive antimicrobials were more frequently administered in the non–β-lactam SAP group (eg, metronidazole: 17.3% vs 11.9%; *P* < .001).

Of the total study population, 9871 patients (2.8%) developed SSIs (Table 2). The incidence of SSI was significantly different between prophylaxis groups, with 9507 patients (2.8%) in the β-lactam SAP group vs 364 patients (6.1%) in the non–β-lactam SAP group (*P* < .001). The non–β-lactam SAP group consistently had higher rates of superficial incisional infections (2.9% vs 1.0%; *P* < .001), deep incisional infections (1.0% vs 0.4%; *P* < .001), and organ-space infections (2.3% vs 1.3%; *P* < .001) (Table 2).

**Table 2.** Crude SSI Rate Per SAP Exposure Group and SSI Type

Type	SSIs, No. (%)		*P* value
	β-lactam SAP	Non–β-lactam SAP	
Overall	9507 (2.8)	364 (6.1)	<.001
Superficial	3559 (1.0)	171 (2.9)	<.001
Deep	1393 (0.4)	57 (1.0)	<.001
Organ-space	4555 (1.3)	136 (2.3)	<.001

Abbreviations: SAP, surgical antimicrobial prophylaxis; SSI, surgical site infections.

The differences in SSI rates were still significant between groups at substance levels: 2.7% for cefazolin and 2.8% for cefuroxime vs 8.0% for ciprofloxacin, 4.1% for vancomycin, and 5.6% for clindamycin (*P* < .001) (eTable 2 in Supplement 1). The identified pathogens stratified by prophylaxis group are detailed in eTable 3 in Supplement 1.

The fully adjusted logistic regression model found that non–β-lactam SAP was independently associated with a higher SSI rate compared with β-lactam SAP (adjusted odds ratio [aOR], 1.78; 95% CI, 1.59-1.99; *P* < .001) (Table 3). Other variables independently associated with a higher SSI rate were an ASA score of 3 to 5 (aOR vs 1-2, 1.73; 95% CI, 1.65-1.81; *P* < .001), clean-contaminated (aOR vs clean wounds, 1.45; 95% CI, 1.13-1.85; *P* < .004) and contaminated (aOR vs clean wounds, 2.06; 95% CI, 1.62-2.64; *P* < .001) surgical wounds, hospital size 500 beds or more (aOR vs <200 beds, 1.38; 95% CI, 1.30-1.46; *P* < .001), and prolonged operative duration exceeding standard procedural times (aOR, 1.53; 95% CI, 1.46-1.60; *P* < .001). Conversely, female sex (aOR, 0.81; 95% CI, 0.77-0.84; *P* < .001) and age 60 years or older (aOR, 0.94; 95% CI, 0.89-0.99; *P* = .01) were associated with lower SSI risk (Table 3).

**Table 3.** Fully Adjusted Mixed-Effects Logistic Regression Models for Risk of Overall Surgical Site Infections^a^

Variable	aOR (95% CI)	*P* value
Non–β-lactam SAP (vs β-lactam SAP)	1.78 (1.59-1.99)	<.001
ASA score 3-5 (vs 1-2)	1.73 (1.65-1.81)	<.001
Wound contamination		
Class I, clean	1 [Reference]	NA
Class II, clean-contaminated	1.45 (1.13-1.85)	<.004
Class III, contaminated	2.06 (1.62-2.64)	<.001
T-time: surgery exceeded standard time (vs surgery within standard time)	1.53 (1.46-1.60)	<.001
Female sex (vs male)	0.81 (0.77-0.84)	<.001
Hospital size		
<200 beds	1 [Reference]	NA
200-499 beds	1.18 (1.12-1.23)	<.001
≥500	1.38 (1.30-1.46)	<.001
Age >60 y (vs <60 y)	0.94 (0.89-0.99)	.01

Abbreviations: aOR, adjusted odds ratio; ASA, American Society of Anesthesiologists; NA, not applicable; SAP, surgical antimicrobial prophylaxis.

^a^ Only complete cases (346 639 of 348 793 patients).

**Figure 2.**
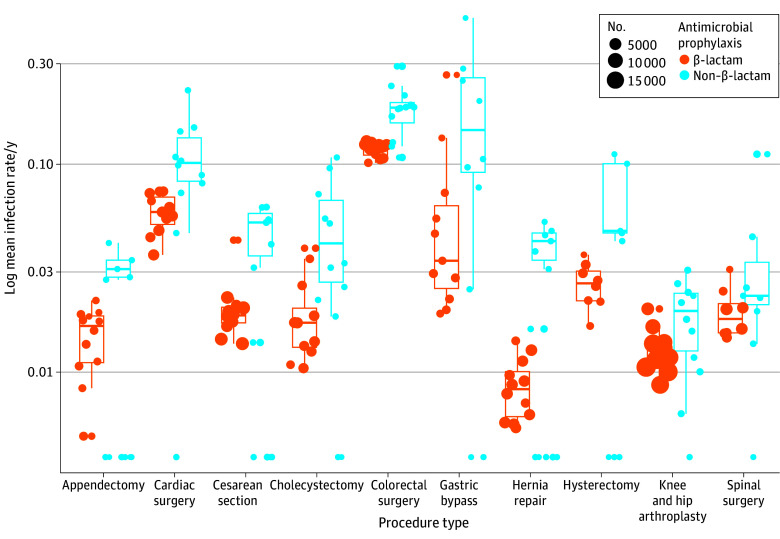
Mean Raw Infection Rate Per Procedure Type for β-Lactam vs Non–β-Lactam Surgical Antimicrobial Prophylaxis

Of importance, non–β-lactam SAP showed higher SSI rates for all surgical procedure types compared with β-lactam SAP (Figure 2). Furthermore non–β-lactam SAP was independently associated with higher rates for all types of SSI, decreasing with depth: superficial incisional SSI (aOR, 2.16; 95% CI, 1.85-2.52; *P* < .001), deep incisional SSI (aOR, 1.74; 95% CI, 1.33-2.26; *P* < .001), and organ space SSI (aOR, 1.34; 95% CI, 1.13-1.60; *P* < .001) (eTable 4 in Supplement 1). The results of multivariable models stratified per wound contamination class are shown in eTable 5 and eTable 6 in Supplement 1, as well as stratified by hospital size in eTable 7 in Supplement 1. The results of the fully adjusted models were in line with the main analysis.

Secondary analyses found significant associations between each non–β-lactam SAP agent and higher SSI risk compared with β-lactam SAP: clindamycin had the highest risk (aOR, 2.12; 95% CI, 1.82-2.47; *P* < .001), followed by ciprofloxacin (aOR, 1.57; 95% CI, 1.33-1.87; *P* < .001) and vancomycin (aOR, 1.38; 95% CI, 1.03-1.86; *P* = .036) (eTable 8 in Supplement 1). Further secondary analyses comparing individual SAP agents to cefazolin are detailed in eTable 9 in Supplement 1. They revealed a higher SSI risk for cefuroxime compared with cefazolin (aOR, 1.16; 95% CI, 1.10-1.22; *P* < .001). The propensity score–based analysis with the 3:1 matched cohort between the SAP groups (within the procedure type clusters) confirmed the significantly higher SSI risk associated with non–β-lactam SAP (aOR, 1.68; 95% CI, 1.47-1.92; *P* < .001) and was well within the 95% CI of the main analysis (eTable 10, eTable 11, and eFigure 3 in Supplement 1).

## Discussion

In this large nationwide cohort study, non–β-lactam SAP was associated with a 1.8-fold higher odds of SSI compared with β-lactam prophylaxis. This association was further established by our propensity score–based and matched analyses, which found 1.7 higher odds of SSI with non–β-lactam prophylaxis.

The increased SSI risk associated with non–β-lactam SAP was consistently observed across all surgical procedure categories, all 3 SSI subtypes, all sizes of hospitals (to exclude confounding by different case mixes), and all individual non–β-lactam agents. In addition, the risk was similar in our stratified analysis for clean-surgery only, as well as surgery with contaminated wounds, arguing against confounding of the overall association by the large proportion of patients with arthroplasty surgery.

These findings are consistent with those of Blumenthal et al,^9^ who found a 50% increased likelihood of SSI in patients with penicillin allergy who received alternative prophylaxis. Our study extends these findings, yielding more definitive results with a much larger sample size and direct comparison of β-lactam vs non–β-lactam prophylaxis, independent of allergy status.

Previous procedure-specific studies examining non–β-lactam SAP and SSI risk have yielded heterogeneous results. Kuriakose et al^11^ reported an increased SSI risk with non–β-lactam SAP in 9949 colectomies (OR, 1.65; 95% CI, 1.20-2.26; *P* < .001), a finding reproduced by Roebke et al^23^ in posterior lumbar fusions among patients with penicillin allergy. A meta-analysis by Lin et al^24^ on 872 patients undergoing colorectal procedures found higher short-term SSI risk with non–β-lactams, but no difference at 30 days. On the other hand, Stone et al^25^ found comparable SSI rates for both types of prophylaxis in 4903 hip and knee arthroplasties.^25^ In contrast to these procedure-specific analyses, our comprehensive study found consistently increased SSI risk with non–β-lactam SAP across all procedure types. Interestingly, the risk was significantly higher for superficial and deep wound infections compared with organ space infections, arguing for a smaller role of SAP choice for organ space infection and possibly a larger role of patient- and surgeon-associated factors.

Beyond their superiority in terms of SSI risk, several additional factors may support the use of β-lactams for SAP. These include considerations on antimicrobial resistance, adverse effects (eg, increased risk for *Clostridium difficile* infection for clindamycin), and the longer half-life times of vancomycin and ciprofloxacin, which require administration more than 60 minutes before incision—whereas β-lactams with shorter half-life times are more easily administered when the patient is already in the operating room.^7,15,26^

Reported penicillin allergy is a common indication for non–β-lactam SAP.^10^ However, as demonstrated in a meta-analysis by Sousa-Pinto et al,^27^ penicillin allergies are frequently unconfirmed, and cross-reactivity with cephalosporins is rare.^27^ Consequently, studies have shown that preoperative allergy testing effectively reduces non–β-lactam prescriptions.^28,29^ These findings coupled with the increased SSI risk associated with non–β-lactam SAP, as confirmed by our study, should motivate preoperative testing in case of uncertain penicillin allergy.

We do not believe that colonization with methicillin-resistant *Staphylococcus aureus* (MRSA) was the main driver for vancomycin administration in our study. The reason for this is that MRSA prevalence overall in Swiss hospitals is relatively low (approximately 7% in 2024).^30^ In our cohort, we only detected 92 SSI events with MRSA as the main or secondary pathogen. In line with the overall prevalence, this represented 8% of all *S. aureus* SSIs. Of interest, none of the 92 patients with MRSA SSI received vancomycin SAP, this points toward the fact, that these cases were incidental findings around SSI detection. The rate of methicillin-resistance among the coagulase-negative staphylococci is approximately 40% across hospitals in Switzerland, and in line with the results of the 2023 study by Peel et al,^31^ additional vancomycin coverage (outside cardiac surgery) is rare in Switzerland.

### Limitations and Strengths

This study has some limitations. First, its retrospective design using a limited number of variables predefined in an SSI surveillance program did not allow capture of important potential confounding factors, like diabetes, perioperative glycaemia, immunosuppression, preoperative infections at remote body sites, preoperative MRSA carriage, preoperative digestive and skin decolonization, nutritional status, intraoperative temperature, intraoperative or postoperative antimicrobial redosing, extended prophylaxis protocols, and specific dosing regimen.^3^ Second, missing information on β-lactam allergy status and institutional antimicrobial guidelines hindered our drawing conclusions about the decision-making process for the administration of non–β-lactam SAP. Third, the inclusion of only 5 specific SAP agents and the exclusion of 129 741 patients who received alternative prophylactic agents or a combination of β-lactam and non–β-lactam agents may affect our study’s generalizability.

Nevertheless, we consider the external validity of this study to be high for comparable health care settings, since hospitals throughout Switzerland participated, ranging from small institutions to large university hospitals. In addition, the robustness of our findings is strengthened by methodological features, including a large sample size, the use of 2 complementary statistical methods, an extended study duration, comprehensive postdischarge surveillance, and a small loss to follow-up (with missing data analyses suggesting random distribution). Another strength of our study consists in the multilevel analysis with clustering at the intervention level, which allowed us to control for potential variation in SSI rate between different procedures. Although the models could not capture all confounding factors, they incorporated key factors, including hospital size and important patient and operation characteristics. Furthermore, the internal validity of this study is high, since it was built on a well-established nationwide surveillance system featuring standardized data collection by trained professionals and regular quality assessment.^13,14^

## Conclusions

In this large cohort study, non–β-lactam SAP was independently associated with 1.8-fold higher odds of SSI across all surgical procedures compared with β-lactam SAP. These findings have important clinical implications, suggesting that β-lactam prophylaxis should be prioritized whenever possible. Patients with self-reported or poorly documented β-lactam allergy should be carefully evaluated before administering a second-line non–β-lactam SAP.

## Notes

Supplement 1. eFigure 1. Overall Age Distribution of Both Male and Female Patients eFigure 2. Timing of Administration of SAP by SAP Exposure Group eTable 1. Missing Data: Comparison of Characteristics of Patients Included and Patients Lost to Follow-up eTable 2. Crude SSI Rate per SAP Type and SSI Type eTable 3. Summary of the Leading Microorganism Detected by SAP Group and Time of Detection eTable 4. Fully Adjusted Mixed Effects Logistic Regression Models with Superficial, Deep and Organ Space SSI as the Dependent Variables eTable 5. Fully Adjusted Mixed Effects Logistic Regression with Overall SSI as the Dependent Variable for the Stratified Analysis (Clean Surgery Only) eTable 6. Fully Adjusted Mixed Effects Logistic Regression with Overall SSI as the Dependent Variable for the Stratified Analysis (Clean-contaminated and Contaminated Surgery Only) eTable 7. Fully Adjusted Mixed Effects Logistic Regression with Overall SSI as the Dependent Variable for the Stratified Analyses by Hospital Size eTable 8. Fully Adjusted Mixed Effects Logistic Regression Models with Overall SSI as the Dependent Variable: Secondary Analysis for Beta-lactam SAP (Cefazolin combined with Cefuroxime) vs the Individual Non-beta-lactam SAP (Ciprofloxacin, Vancomycin, Clindamycin) eTable 9. Fully Adjusted Mixed Effects Logistic Regression Models with Overall SSI as the Dependent Variable: Secondary Analysis for Cefazolin vs the Other Individual SAP (Cefuroxime, Ciprofloxacin, Vancomycin, Clindamycin) eTable 10. Propensity Score Statistics (before / after 3:1 Matching) in the Beta-Lactam and Non-Beta-Lactam SAP Group (example within the colorectal clusters) eFigure 3. Distribution of Propensity Scores before / after 3:1 Matching in the Beta-Lactam and Non-Beta-Lactam SAP Group (example within the colorectal clusters) eTable 11. Mixed Effect Logistic Regression Results for SSI after 3:1 Propensity Score Matching in the Procedure Type Clusters

Supplement 2. Nonauthor Members of the Swissnoso Group

Supplement 3. Data Sharing Statement
