# Supplementary material for: β-Lactam vs Non–β-Lactam Antimicrobial Prophylaxis and Surgical Site Infection
Source: JAMA Netw Open. 2025 Oct 31;8(10):e2540809. doi: 10.1001/jamanetworkopen.2025.40809 (PMC12579348; doi:10.1001/jamanetworkopen.2025.40809)
Supplement: Supplement 1. — eFigure 1. Overall Age Distribution of Both Male and Female Patients eFigure 2. Timing of Administration of SAP by SAP Exposure Group eTable 1. Missing Data: Comparison of Characteristics of Patients Included and Patients Lost to Follow-up eTable 2. Crude SSI Rate per SAP Type and SSI Type eTable 3. Summary of the Leading Microorganism Detected by SAP Group and Time of Detection eTable 4. Fully Adjusted Mixed Effects Logistic Regression Models with Superficial, Deep and Organ Space SSI as the Dependent Variables eTable 5. Fully Adjusted Mixed Effects Logistic Regression with Overall SSI as the Dependent Variable for the Stratified Analysis (Clean Surgery Only) eTable 6. Fully Adjusted Mixed Effects Logistic Regression with Overall SSI as the Dependent Variable for the Stratified Analysis (Clean-contaminated and Contaminated Surgery Only) eTable 7. Fully Adjusted Mixed Effects Logistic Regression with Overall SSI as the Dependent Variable for the Stratified Analyses by Hospital Size eTable 8. Fully Adjusted Mixed Effects Logistic Regression Models with Overall SSI as the Dependent Variable: Secondary Analysis for Beta-lactam SAP (Cefazolin combined with Cefuroxime) vs the Individual Non-beta-lactam SAP (Ciprofloxacin, Vancomycin, Clindamycin) eTable 9. Fully Adjusted Mixed Effects Logistic Regression Models with Overall SSI as the Dependent Variable: Secondary Analysis for Cefazolin vs the Other Individual SAP (Cefuroxime, Ciprofloxacin, Vancomycin, Clindamycin) eTable 10. Propensity Score Statistics (before / after 3:1 Matching) in the Beta-Lactam and Non-Beta-Lactam SAP Group (example within the colorectal clusters) eFigure 3. Distribution of Propensity Scores before / after 3:1 Matching in the Beta-Lactam and Non-Beta-Lactam SAP Group (example within the colorectal clusters) eTable 11. Mixed Effect Logistic Regression Results for SSI after 3:1 Propensity Score Matching in the Procedure Type Clusters [file jamanetwopen-e2540809-s001.pdf]

Supplemental Online Content

Largiadèr S, Berthod D, Widmer A, et al; SWISSNOSO. β-Lactam vs non–β-lactam antimicrobial prophylaxis and surgical site infection. *JAMA Netw Open*. 2025;8(10):e2540809. doi:10.1001/jamanetworkopen.2025.40809

eFigure 1. Overall Age Distribution of Both Male and Female Patients
eFigure 2. Timing of Administration of SAP by SAP Exposure Group
eTable 1. Missing Data: Comparison of Characteristics of Patients Included and Patients Lost to Follow-up
eTable 2. Crude SSI Rate per SAP Type and SSI Type
eTable 3. Summary of the Leading Microorganism Detected by SAP Group and Time of Detection
eTable 4. Fully Adjusted Mixed Effects Logistic Regression Models with Superficial, Deep and Organ Space SSI as the Dependent Variables
eTable 5. Fully Adjusted Mixed Effects Logistic Regression with Overall SSI as the Dependent Variable for the Stratified Analysis (Clean Surgery Only)
eTable 6. Fully Adjusted Mixed Effects Logistic Regression with Overall SSI as the Dependent Variable for the Stratified Analysis (Clean-contaminated and Contaminated Surgery Only)
eTable 7. Fully Adjusted Mixed Effects Logistic Regression with Overall SSI as the Dependent Variable for the Stratified Analyses by Hospital Size
eTable 8. Fully Adjusted Mixed Effects Logistic Regression Models with Overall SSI as the Dependent Variable: Secondary Analysis for Beta-lactam SAP (Cefazolin combined with Cefuroxime) vs the Individual Non-beta-lactam SAP (Ciprofloxacin, Vancomycin, Clindamycin)
eTable 9. Fully Adjusted Mixed Effects Logistic Regression Models with Overall SSI as the Dependent Variable: Secondary Analysis for Cefazolin vs the Other Individual SAP (Cefuroxime, Ciprofloxacin, Vancomycin, Clindamycin)
eTable 10. Propensity Score Statistics (before / after 3:1 Matching) in the Beta-Lactam and Non-Beta-Lactam SAP Group (example within the colorectal clusters)
eFigure 3. Distribution of Propensity Scores before / after 3:1 Matching in the Beta-Lactam and Non-Beta-Lactam SAP Group (example within the colorectal clusters)
eTable 11. Mixed Effect Logistic Regression Results for SSI after 3:1 Propensity Score Matching in the Procedure Type Clusters

This supplemental material has been provided by the authors to give readers additional information about their work.

© 2025 Largiadèr S et al. *JAMA Network Open*.

eFigure 1. Overall Age Distribution of Both Male and Female Patients

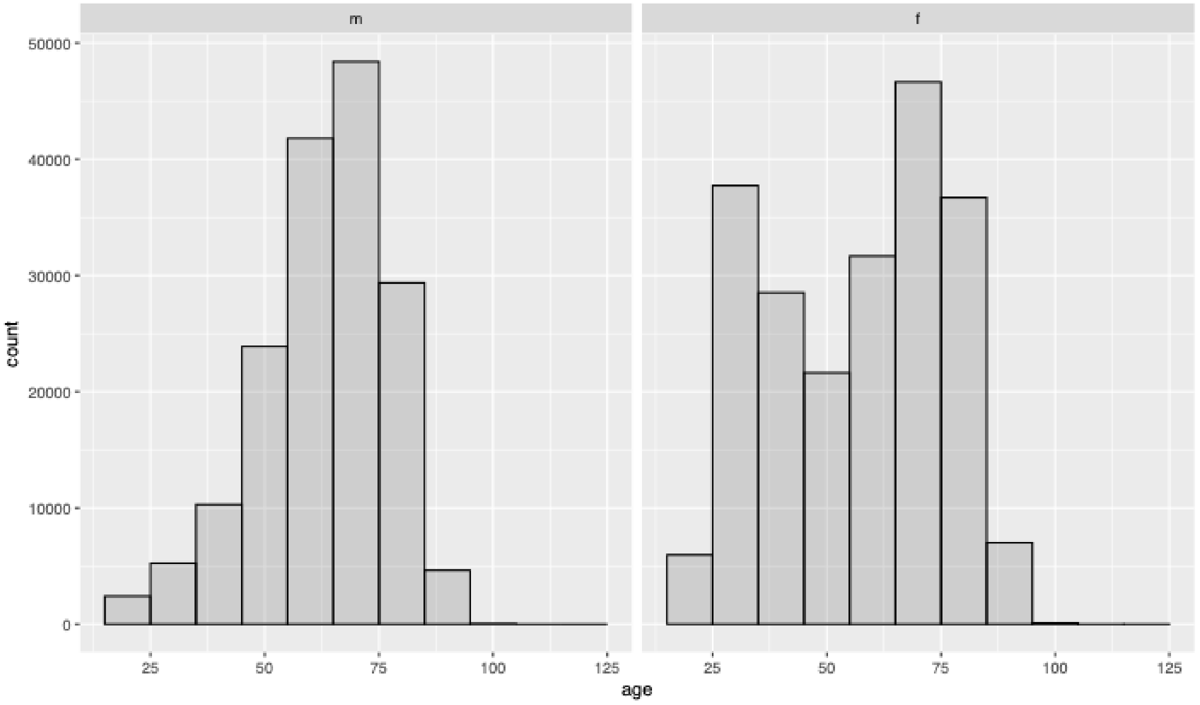

Abbreviations:

m	male
f	female

eFigure 2. Timing of Administration of SAP (Prior to Incision) by SAP Exposure Group

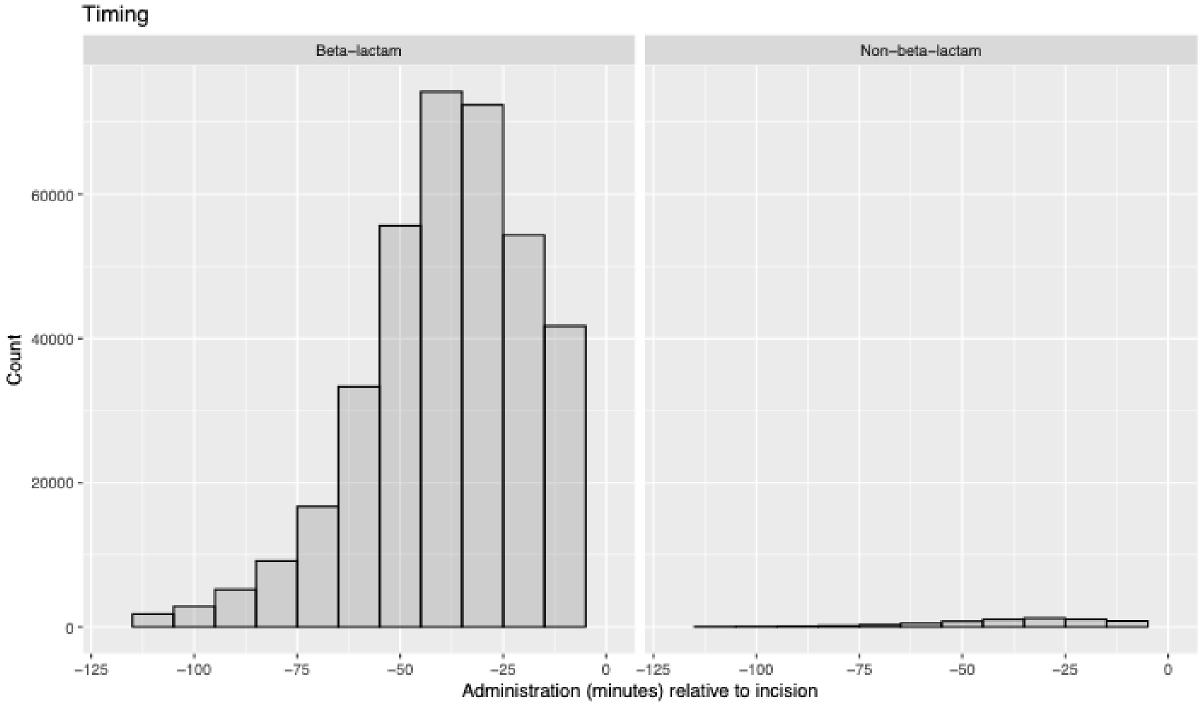

Abbreviations:
SAP Surgical Antimicrobial Prophylaxis

© 2025 Largiadèr S et al. *JAMA Network Open*.

eTable 1. Missing Data: Comparison of Characteristics of Patients Included and Patients Lost to Follow-up

Characteristic	Patients included	Patients lost to follow-up	*P* value
N	348 885	33 233	
Non-beta-lactam SAP (%)	5 949 (1.7)	464 (1.4)	<0.001
Age (median [IQR])	63.15 [47.03, 73.26]	59.69 [41.65, 71.69]	<0.001
Sex = female (%)	196 411 (56.3)	19 580 (58.9)	<0.001
ASA score = 3-5 (%)	97 730 (28.0)	9 770 (29.7)	<0.001
Wound contamination class (%)			0.185
Class I (clean)	222 632 (63.8)	21 151 (63.6)	
Class II (clean-contaminated)	99 383 (28.5)	9 592 (28.9)	
Class III (contaminated)	26 870 (7.7)	2 490 (7.5)	
Surgery exceeded standard time (%)	60 062 (17.2)	5 229 (15.7)	<0.001
Year of procedure (median [IQR])	2015 [2013, 2018]	2015 [2012, 2018]	<0.001
Hospital size (beds, %)			<0.001
<200	191 192 (54.8)	19 668 (59.2)	
200-499	100 957 (28.9)	8 414 (25.3)	
500+	56 736 (16.3)	5 151 (15.5)	
Endoscopic surgery			<0.001
No	233 793 (67.0)	23 617 (71.1)	
Yes	110 492 (31.7)	9 316 (28.0)	
Beginning as endoscopy	4 131 (1.2)	249 (0.7)	
Transvaginal procedure	361 (0.1)	29 (0.1)	
Transanal procedure	30 (0.0)	16 (0.0)	
NA	78 (0.0)	6 (0.0)	
Implant	210 883 (60.4)	20 500 (61.7)	<0.001
Procedure Type (%)			<0.001
Appendectomy	7 089 (2.0)	707 (2.1)	
Cardiac surgery	25 116 (7.2)	3 576 (10.8)	
Caesarean delivery	46 350 (13.3)	5 788 (17.4)	
Cholecystectomy	21 877 (6.3)	1 902 (5.7)	
Colorectal surgery	34 099 (9.8)	1 941 (5.8)	
Gastric bypass surgery	7 079 (2.0)	601 (1.8)	
Hernia repair	32 028 (9.2)	2 214 (6.7)	
Hysterectomy	8 183 (2.3)	991 (3.0)	
Knee and Hip Arthroplasty	150 072 (43.0)	14 314 (43.1)	
Spinal surgery (Laminectomy, Spinal fusion)	16 992 (4.9)	1 199 (3.6)	
Elective surgery (%)	301 353 (86.4)	28 105 (84.6)	<0.001
Choice of primary SAP (%)			<0.001
Cefazolin	85 523 (24.5)	6 610 (19.9)	
Cefuroxime	257 413 (73.8)	26 159 (78.7)	
Ciprofloxacin	1 813 (0.5)	144 (0.4)	
Vancomycin	949 (0.3)	73 (0.2)	
Clindamycin	3 187 (0.9)	247 (0.7)	
Addition of second SAP (%)			<0.001
Gentamicin	77 (0.0)	4 (0.0)	
Metronidazole	41 923 (12.0)	2 706 (8.1)	
NA	306 885 (88.0)	30 523 (91.8)	
Addition of third SAP (%)			0.030
Gentamicin	20 (0.0)	0 (0.0)	
Metronidazole	92 (0.0)	2 (0.0)	
NA	348 773 (100.0)	33 231 (100.0)	

Abbreviations:

SAP	Surgical Antimicrobial Prophylaxis
IQR	Interquartile range
ASA	American Society of Anesthesiologists´ Score
NA	not available

© 2025 Largiadèr S et al. *JAMA Network Open*.

eTable 2. Crude SSI Rate per SAP Type and SSI Type

SSI type	Cefazolin	Cefuroxime	Ciprofloxacin	Vancomycin	Clindamycin	*P* value
N	85 523	257 413	1 813	949	3 187	
Overall SSI (%)	2 323 (2.7)	7 184 (2.8)	145 (8.0)	39 (4.1)	180 (5.6)	<0.001
Superficial SSI (%)	871 (1.0)	2 688 (1.0)	72 (4.0)	19 (2.0)	80 (2.5)	<0.001
Deep SSI (%)	320 (0.4)	1 073 (0.4)	15 (0.8)	6 (0.6)	36 (1.1)	<0.001
Organ-space SSI (%)	1 132 (1.3)	3 423 (1.3)	58 (3.2)	14 (1.5)	64 (2.0)	<0.001

Abbreviations:
SAP Surgical Antimicrobial Prophylaxis
SSI Surgical Site Infections

© 2025 Largiadèr S et al. *JAMA Network Open*.

eTable 3. Summary of the Leading Microorganism Detected by SAP Group and Time of Detection

**Microbiologic etiology**	**Detected after 30d FUP**		**Detected after 1y FUP**	
SAP Exposure Group	*Beta-lactam, No (%)*	*Non-beta-lactam, No (%)*	*Beta-lactam, No (%)*	*Non-beta-lactam, No (%)*
**Gram positive**				
Methicillin-susceptible Staphylococcus aureus	527 (12.7)	11 (6.1)	395 (26.5)	4 (9.1)
Methicillin-resistant Staphylococcus aureus	36 (0.9)	4 (2.2)	34 (2.3)	1 (2.3)
Coagulase-negative Staphylococcus	472 (11.4)	24 (13.3)	522 (35.0)	22 (50.0)
Streptococcus pneumoniae	2 (0.0)	1 (0.6)	1 (0.1)	0 (0.0)
Enterococcus faecium (non VRE)	105 (2.5)	4 (2.2)	6 (0.4)	0 (0.0)
Enterococcus faecalis and other Enterococci (non VRE)	129 (3.1)	5 (2.8)	14 (0.9)	1 (2.3)
VRE	9 (0.2)	0 (0.0)	0 (0.0)	0 (0.0)
Alpha-haemolytic Streptococcus	127 (3.1)	8 (4.4)	8 (0.5)	0 (0.0)
Streptococcus pyogenes	10 (0.2)	1 (0.6)	3 (0.2)	0 (0.0)
Streptococcus agalactiae	70 (1.7)	3 (1.7)	25 (1.7)	0 (0.0)
Other streptococcus	73 (1.8)	2 (1.1)	34 (2.3)	0 (0.0)
Bacillus sp.	5 (0.1)	0 (0.0)	2 (0.1)	0 (0.0)
Corynebacterium sp.	14 (0.3)	1 (0.6)	7 (0.5)	0 (0.0)
Other Gram positive	32 (0.8)	2 (1.1)	5 (0.3)	0 (0.0)
**Gram negative**				
Escherichia coli	902 (21.7)	45 (25.0)	61 (4.1)	2 (4.5)
ESBL-producing Escherichia coli	151 (3.6)	4 (2.2)	7 (0.5)	1 (2.3)
Carbapenemase-producing Escherichia coli	2 (0.0)	0 (0.0)	0 (0.0)	0 (0.0)
Klebsiella pneumoniae, oxytoca, variicola	117 (2.8)	10 (5.6)	34 (2.3)	0 (0.0)
ESBL-producing Klebsiella sp.	15 (0.4)	0 (0.0)	1 (0.1)	0 (0.0)
Proteus mirabilis, vulgaris	69 (1.7)	3 (1.7)	24 (1.6)	2 (4.5)
Serratia marcescens	33 (0.8)	2 (1.1)	25 (1.7)	1 (2.3)
Carbapenemase-producing Serratia marcescens	1 (0.0)	0 (0.0)	0 (0.0)	0 (0.0)
ESBL-producing Serratia marcescens	1 (0.0)	0 (0.0)	0 (0.0)	0 (0.0)
Enterobacter aerogenes / cloacae	158 (3.8)	2 (1.1)	53 (3.6)	1 (2.3)
Carbapenemase-producing Enterobacter aerogenes / cloacae	2 (0.0)	0 (0.0)	3 (0.2)	0 (0.0)
ESBL-producing Enterobacter sp.	1 (0.0)	0 (0.0)	0 (0.0)	0 (0.0)
Other Enterobacteriaceae	159 (3.8)	4 (2.2)	23 (1.5)	1 (2.3)
Carbapenemase-producing other Enterobacteriaceae	1 (0.0)	0 (0.0)	0 (0.0)	0 (0.0)
Pseudomonas aeruginosa	183 (4.4)	5 (2.8)	47 (3.2)	2 (4.5)
Pseudomonas non aeruginosa	2 (0.0)	0 (0.0)	1 (0.1)	0 (0.0)
Acinetobacter sp.	9 (0.2)	0 (0.0)	2 (0.1)	0 (0.0)
Haemophilus sp.	6 (0.1)	0 (0.0)	1 (0.1)	0 (0.0)
Stenotrophomonas maltophilia	3 (0.1)	0 (0.0)	1 (0.1)	0 (0.0)
Other Gram negatives	6 (0.1)	1 (0.6)	3 (0.2)	0 (0.0)

© 2025 Largiadèr S et al. *JAMA Network Open*.

**Anaerobic**				
Cutibacterium acnes	92 (2.2)	3 (1.7)	82 (5.5)	2 (4.5)
Clostridium perfringens	3 (0.1)	1 (0.6)	1 (0.1)	0 (0.0)
Clostridium species	9 (0.2)	1 (0.6)	0 (0.0)	0 (0.0)
Peptostreptococcus sp.	16 (0.4)	0 (0.0)	7 (0.5)	1 (2.3)
Prevotella sp.	18 (0.4)	0 (0.0)	0 (0.0)	0 (0.0)
Bacteroïdes sp.	97 (2.3)	3 (1.7)	1 (0.1)	0 (0.0)
Fusobacterium	2 (0.0)	0 (0.0)	0 (0.0)	0 (0.0)
Actinomyces	3 (0.1)	1 (0.6)	1 (0.1)	0 (0.0)
Veillonellae	3 (0.1)	0 (0.0)	0 (0.0)	0 (0.0)
Other Anaerobic	32 (0.8)	3 (1.7)	9 (0.6)	2 (4.5)
**Fungi**				
Candida albicans	71 (1.7)	5 (2.8)	3 (0.2)	0 (0.0)
Candida glabrata	11 (0.3)	0 (0.0)	1 (0.1)	0 (0.0)
Other Candida sp.	4 (0.1)	0 (0.0)	0 (0.0)	0 (0.0)
Other fungi	3 (0.1)	0 (0.0)	0 (0.0)	0 (0.0)
**Non-classifiable**	18 (0.4)	1 (0.6)	2 (0.1)	0 (0.0)
**Other / not identified**	339	2	41	1
**NA**	338 744	5 768	341 393	5 901

Abbreviations:

SAP	Surgical Antimicrobial Prophylaxis
FUP	Follow-up
Sp.	Species
NA	not available

© 2025 Largiadèr S et al. *JAMA Network Open*.

eTable 4. Fully Adjusted Mixed Effects Logistic Regression Models with Superficial, Deep and Organ Space SSI as the Dependent Variables

**Variable**	**aOR and 95% CI**	***P* value**
Superficial Incisional SSI		
Non-beta-lactam SAP (Ref: Beta-lactam SAP)	2.16 (1.85-2.52)	<0.001
ASA score: 3-5 (Ref: ASA score 1-2)	1.59 (1.47-1.73)	<0.001
Wound contamination class II, clean-contaminated (Ref: wound contamination class I, clean)	1.11 (0.82-1.50)	0.491
Wound contamination class III, contaminated (Ref: wound contamination class I, clean)	1.48 (1.09-2.01)	0.0119
T-time: surgery exceeded standard time (Ref: surgery within standard time)	1.37 (1.27-1.47)	<0.001
Sex: female (Ref: male)	0.95 (0.88-1.02)	0.153
Hospital size: 200-499 beds (Ref: <200 beds)	1.24 (1.14-1.34)	<0.001
Hospital size: 500+ beds (Ref: <200 beds)	1.27 (1.16-1.39)	<0.001
Age category: >60y (Ref: <60y)	0.84 (0.77-0.91)	<0.001
Deep Incisional SSI		
Non-beta-lactam SAP (Ref: Beta-lactam SAP)	1.74 (1.33-2.26)	<0.001
ASA score: 3-5 (Ref: ASA score 1-2)	1.86 (1.63-2.12)	<0.001
Wound contamination class II, clean-contaminated (Ref: wound contamination class I, clean)	2.07 (1.34-3.20)	>=0.001
Wound contamination class III, contaminated (Ref: wound contamination class I, clean)	3.06 (1.98-4.73)	<0.001
T-time: surgery exceeded standard time (Ref: surgery within standard time)	1.63 (1.45-1.83)	<0.001
Sex: female (Ref: male)	0.98 (0.88-1.11)	0.812
Hospital size: 200-499 beds (Ref: <200 beds)	1.45 (1.27-1.65)	<0.001
Hospital size: 500+ beds (Ref: <200 beds)	1.62 (1.40-1.88)	<0.001
Age category: >60y (Ref: <60y)	0.98 (0.86-1.11)	0.763
Organ Space SSI		
Non-beta-lactam SAP (Ref: Beta-lactam SAP)	1.34 (1.13-1.60)	<0.001
ASA score: 3-5 (Ref: ASA score 1-2)	1.71 (1.60-1.82)	<0.001
Wound contamination class II, clean-contaminated (Ref: wound contamination class I, clean)	1.46 (1.01-2.10)	0.0435
Wound contamination class III, contaminated (Ref: wound contamination class I, clean)	2.07 (1.43-2.99)	<0.001
T-time: surgery exceeded standard time (Ref: surgery within standard time)	1.55 (1.45-1.65)	<0.001
Sex: female (Ref: male)	0.69 (0.65-0.74)	<0.001
Hospital size: 200-499 beds (Ref: <200 beds)	1.06 (0.99-1.14)	0.108
Hospital size: 500+ beds (Ref: <200 beds)	1.37 (1.27-1.48)	<0.001
Age category: >60y (Ref: <60y)	1.00 (0.93-1.08)	0.951

Abbreviations:

aOR	Adjusted odds ratio
CI	Confidence Interval
SSI	Surgical Site Infections
SAP	Surgical Antimicrobial Prophylaxis
ASA	American Society of Anesthesiologists´ Score
Ref:	Reference value

© 2025 Largiadèr S et al. *JAMA Network Open*.

eTable 5. Fully Adjusted Mixed Effects Logistic Regression with Overall SSI as the Dependent Variable for the Stratified Analysis (Clean Surgery Only, N=222 632)

Only complete cases (221 658 / 222 632).

Variable	aOR and 95% CI	*P* value
Non-beta-lactam SAP (Ref: Beta-lactam SAP)	1.77 (1.47-2.15)	<0.001
ASA score: 3-5 (Ref: ASA score 1-2)	1.89 (1.74-2.05)	<0.001
T-time: surgery exceeded standard time (Ref: surgery within standard time)	1.74 (1.61-1.88)	<0.001
Sex: female (Ref: male)	0.86 (0.80-0.92)	<0.001
Hospital size: 200-499 beds (Ref: <200 beds)	1.12 (1.03-1.22)	0.0064
Hospital size: 500+ beds (Ref: <200 beds)	1.34 (1.22-1.47)	<0.001
Age category: >60y (Ref: <60y)	0.88 (0.81-0.95)	<0.001

Abbreviations

aOR	Adjusted odds ratio
CI	Confidence Interval
SAP	Surgical Antimicrobial Prophylaxis
ASA	American Society of Anesthesiologists score
Ref:	Reference value

© 2025 Largiadèr S et al. *JAMA Network Open*.

eTable 6. Fully Adjusted Mixed Effects Logistic Regression with Overall SSI as the Dependent Variable for the Stratified Analysis (Clean-contaminated and Contaminated Surgery Only, N=126 253)

Only complete cases (125 055 / 126 253).

Variable	aOR and 95% CI	*P* value
Non-beta-lactam SAP (Ref: Beta-lactam SAP)	1.80 (1.57-2.06)	<0.001
ASA score: 3-5 (Ref: ASA score 1-2)	1.63 (1.54-1.74)	<0.001
T-time: surgery exceeded standard time (Ref: surgery within standard time)	1.43 (1.35-1.51)	<0.001
Sex: female (Ref: male)	0.76 (0.72-0.81)	<0.001
Hospital size: 200-499 beds (Ref: <200 beds)	1.21 (1.14-1.29)	<0.001
Hospital size: 500+ beds (Ref: <200 beds)	1.41 (1.31-1.51)	<0.001
Age category: >60y (Ref: <60y)	0.98 (0.92-1.05)	0.559

Abbreviations

aOR	Adjusted odds ratio
CI	Confidence Interval
SAP	Surgical Antimicrobial Prophylaxis
ASA	American Society of Anesthesiologists score
Ref:	Reference value

© 2025 Largiadèr S et al. *JAMA Network Open*.

eTable 7. Fully Adjusted Mixed Effects Logistic Regression with Overall SSI as the Dependent Variable for the Stratified Analyses by Hospital Size

Variable	aOR and 95% CI	*P* value
Hospital size: <200 beds *(n=191 192 patients; full model = 189 747 patients*)*		
Non-beta-lactam SAP (Ref: Beta-lactam SAP)	1.65 (1.38-1.98)	<0.001
Hospital size: 200-499 beds *(n=100 957 patients; full model = 100 377 patients*)*		
Non-beta-lactam SAP (Ref: Beta-lactam SAP)	1.78 (1.41-2.24)	<0.001
Hospital size: >500 beds *(n=56 736 patients; full model = 56 580 patients*)*		
Non-beta-lactam SAP (Ref: Beta-lactam SAP)	1.75 (1.46-2.10)	<0.001

*Full models adjusted for: ASA Score, wound contamination class, T-Score, Age-category, gender (as fixed effects), procedure-type (as random effect)

Abbreviations

aOR	Adjusted odds ratio
CI	Confidence Interval
SAP	Surgical Antimicrobial Prophylaxis

© 2025 Largiadèr S et al. *JAMA Network Open*.

eTable 8. Fully Adjusted Mixed Effects Logistic Regression Models with Overall Surgical Site Infections as the Dependent Variable: Secondary Analysis for Beta-lactam SAP (Cefazolin combined with Cefuroxime) vs the Individual Non-beta-lactam SAP (Ciprofloxacin, Vancomycin, Clindamycin)

Only complete cases (346 639 / 348 793).

**Variable**	**aOR and 95% CI**	***P* value**
Ciprofloxacin SAP (Ref: Cefazolin + Cefuroxime SAP)	1.57 (1.33-1.87)	<0.001
Vancomycin SAP (Ref: Cefazolin + Cefuroxime SAP)	1.38 (1.03-1.86)	0.036
Clindamycin SAP (Ref: Cefazolin + Cefuroxime SAP)	2.12 (1.82-2.47)	<0.001
ASA score: 3-5 (Ref: ASA score 1-2)	1.73 (1.65-1.82)	<0.001
Wound contamination class II, clean-contaminated (Ref: wound contamination class I, clean)	1.46 (1.17-1.82)	<0.001
Wound contamination class III, contaminated (Ref: wound contamination class I, clean)	2.09 (1.67-2.60)	<0.001
T-time: surgery exceeded standard time (Ref: surgery within standard time)	1.52 (1.46-1.60)	<0.001
Sex: female (Ref: male)	0.80 (0.77-0.84)	<0.001
Hospital size: 200-499 beds (Ref: <200 beds)	1.17 (1.12-1.23)	<0.001
Hospital size: 500+ beds (Ref: <200 beds)	1.38 (1.30-1.46)	<0.001
Age category: >60y (Ref: <60y)	0.94 (0.89-0.99)	0.012

Abbreviations:

aOR	Adjusted odds ratio
CI	Confidence Interval
SAP	Surgical Antimicrobial Prophylaxis
ASA	American Society of Anesthesiologists´ Score
Ref:	Reference value

© 2025 Largiadèr S et al. *JAMA Network Open*.

eTable 9. Fully Adjusted Mixed Effects Logistic Regression Models with Overall Surgical Site Infections as the Dependent Variable: Secondary Analysis for Cefazolin vs the Other Individual SAP (Cefuroxime, Ciprofloxacin, Vancomycin, Clindamycin)

Only complete cases (346 639 / 348 793).

Variable	aOR and 95% CI	*P* value
Cefuroxime SAP (Ref: Cefazolin SAP)	1.16 (1.10-1.22)	<0.001
Ciprofloxacin SAP (Ref: Cefazolin SAP)	1.75 (1.46-2.09)	<0.001
Vancomycin SAP (Ref: Cefazolin SAP)	1.55 (1.12-2.14)	0.007
Clindamycin SAP (Ref: Cefazolin SAP)	2.36 (2.01-2.73)	<0.001
ASA score: 3-5 (Ref: ASA score 1-2)	1.72 (1.64-1.81)	<0.001
Wound contamination class II, clean-contaminated (Ref: wound contamination class I, clean)	1.46 (1.14-1.87)	0.002
Wound contamination class III, contaminated (Ref: wound contamination class I, clean)	2.09 (1.63-2.67)	<0.001
T-time: surgery exceeded standard time (Ref: surgery within standard time)	1.54 (1.47-1.62)	<0.001
Sex: female (Ref: male)	0.80 (0.77-0.84)	<0.001
Hospital size: 200-499 beds (Ref: <200 beds)	1.17 (1.11-1.23)	<0.001
Hospital size: 500+ beds (Ref: <200 beds)	1.40 (1.32-1.48)	<0.001
Age category: >60y (Ref: <60y)	0.94 (0.89-0.99)	0.017

Abbreviations

aOR	Adjusted odds ratio
CI	Confidence Interval
SAP	Surgical Antimicrobial Prophylaxis
ASA	American Society of Anesthesiologists score
Ref:	Reference value

eTable 10. Propensity Score Statistics (before / after 3:1 Matching) in the Beta-lactam and Non-beta-lactam SAP Group (example within the colorectal clusters)

We used manual 3:1 next neighbour matching in with a maximum caliper of 0.1. This function was re-iterated across all 10 procedure type groups, as matching in between the clusters does not make sense due to different fixed characteristics associated with one procedure type (for example: younger patients, female gender for caesarean section; additional anaerobic coverage for colorectal surgery).

To create our propensity model we used logistic regression, including all clinical and epidemiological relevant covariates across the entire cohort, that could confound the results:

```
ps_model <- glm(a1bin ~ class + age_cat + A + hosp_size,
                data = cluster_data,
                family = binomial)
```

We created detailed analytic functions, the check propensity score statistics within the procedure type clusters.

Here, we present (exemplary) the covariate differences and overall statistics within the cluster “colorectal sugery”. The statistics for the other procedure type clusters and/or the detailed codes are available on request.

```
1. COVARIATE DIFFERENCES BEFORE AND AFTER MATCHING
==================================================
Original Cluster Data:
- Total observations: 33981
- Treated (a1bin=1): 887
- Controls (a1bin=0): 33094

Matched data found
- Total observations: 3548
- Treated (a1bin=1): 887
- Controls (a1bin=0): 2661

MATCHING EFFICIENCY:
- Original treated patients: 887
- Successfully matched treated patients: 887
- Matching rate: 100 %

COVARIATE: CLASS
------------------------
BEFORE MATCHING:
  Treated distribution: I. sauber = 0, II. sauber-kontaminiert = 720, III. kontaminiert = 167
  Control distribution: I. sauber = 0, II. sauber-kontaminiert = 27733, III. kontaminiert = 5361
AFTER MATCHING:
  Treated distribution: I. sauber = 0, II. sauber-kontaminiert = 720, III. kontaminiert = 167
  Control distribution: I. sauber = 0, II. sauber-kontaminiert = 2160, III. kontaminiert = 501

COVARIATE: AGE_CAT
```

BEFORE MATCHING:
  Treated distribution: <60y = 277, >60y = 610
  Control distribution: <60y = 9840, >60y = 23254
AFTER MATCHING:
  Treated distribution: <60y = 277, >60y = 610
  Control distribution: <60y = 831, >60y = 1830

COVARIATE: A

BEFORE MATCHING:
  Treated distribution: 1/2 = 520, 3-5 = 367
  Control distribution: 1/2 = 20907, 3-5 = 12187
AFTER MATCHING:
  Treated distribution: 1/2 = 520, 3-5 = 367
  Control distribution: 1/2 = 1560, 3-5 = 1101

COVARIATE: HOSP_SIZE

BEFORE MATCHING:
  Treated distribution: <200 = 490, 200-499 = 206, 500+ = 191
  Control distribution: <200 = 12452, 200-499 = 13857, 500+ = 6785
AFTER MATCHING:
  Treated distribution: <200 = 490, 200-499 = 206, 500+ = 191
  Control distribution: <200 = 1470, 200-499 = 618, 500+ = 573

	**Before Matching**	**After matching**
**Treated (mean PS. score, SD)**	0.031 , SD: 0.011	0.031 , SD: 0.011
**Controls (mean PS. score, SD**	0.026 , SD: 0.011	0.031 , SD: 0.011
**SMD PS Scores**	0.442	0

Abbreviations
PS Propensity Score
SD Standard deviation
SMD PS Standardized Mean Difference Propensity Score

© 2025 Largiadèr S et al. *JAMA Network Open*.

eFigure 3. Distribution of Propensity Scores before or after 3:1 Matching in the Beta-lactam and Non-beta-lactam SAP Group (example within the colorectal cluster)

Control = Beta-lactam; Treated = Non-beta-lactam

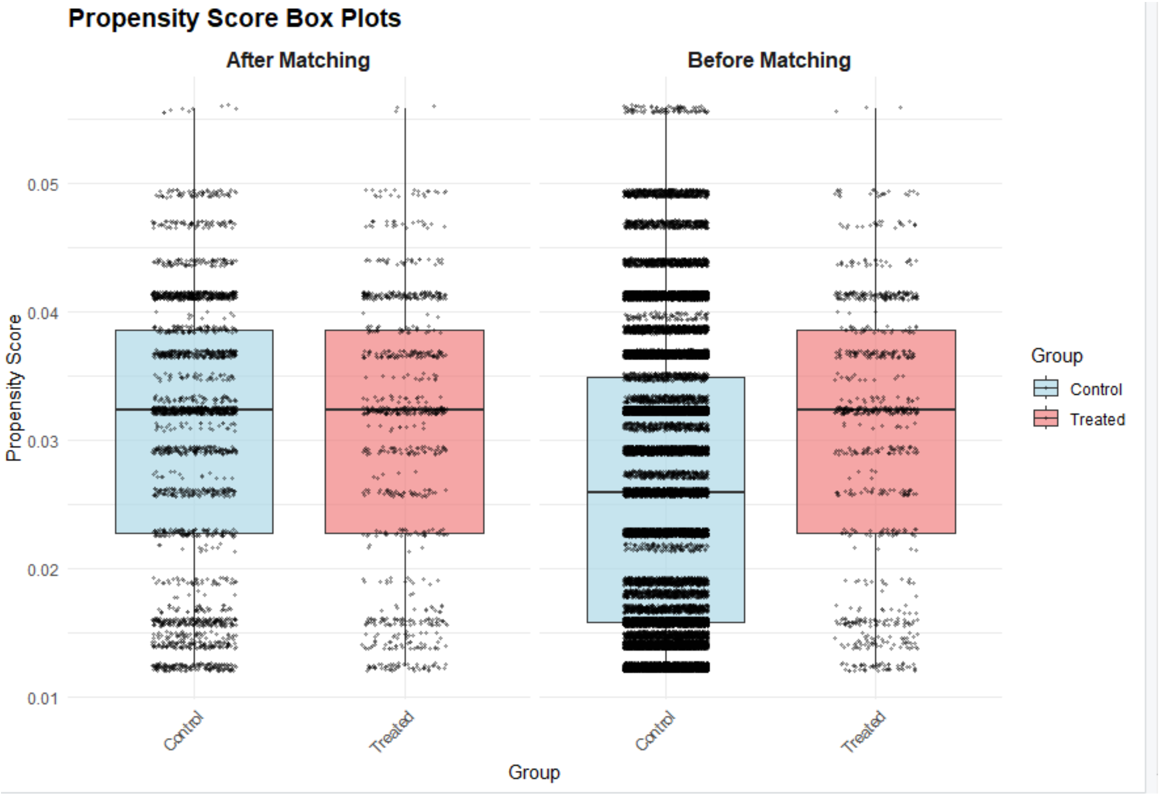

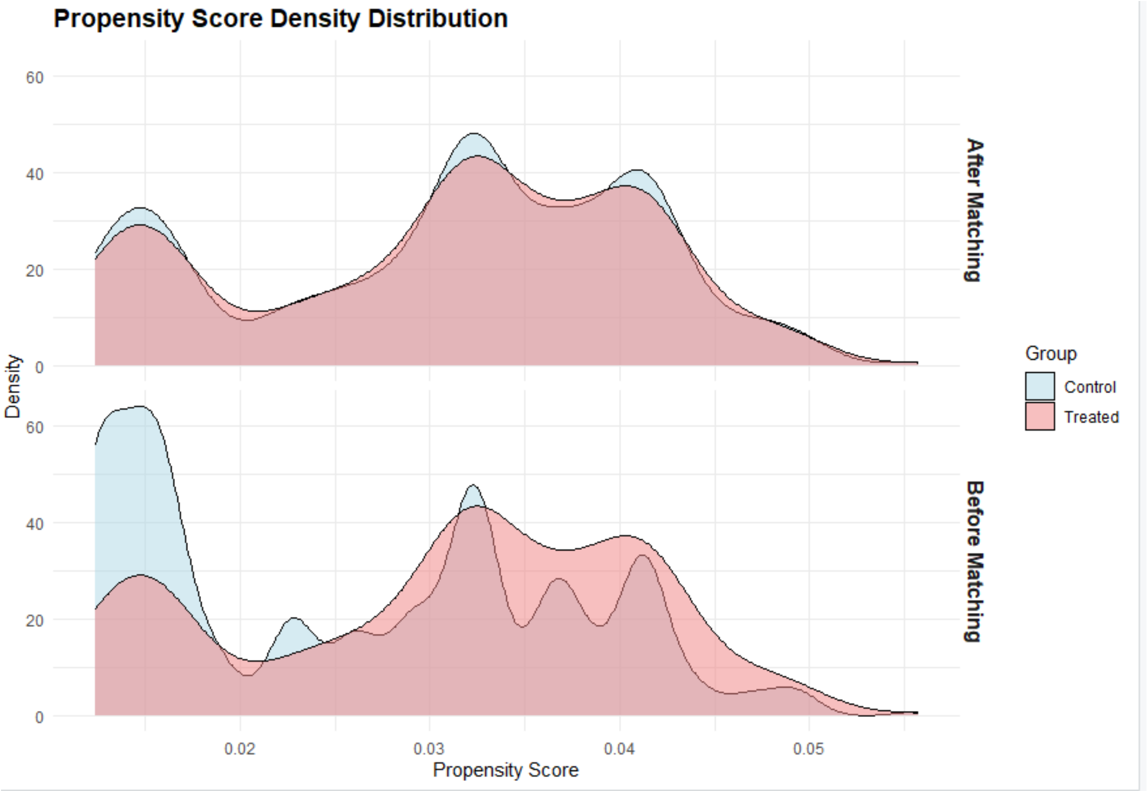

© 2025 Largiadèr S et al. *JAMA Network Open*.

eTable 11. Mixed Effect Logistic Regression Results for SSI After 3:1 Propensity Score Matching in the Procedure Type Clusters

Model with 100% successful 3:1 matches, n=23’700 patients within 10 procedure type clusters
Formula: g<-glmer(inf~exposure+(1|cluster)+ wound_cont_class + age_cat + ASA_score_cat + hosp_size, data=all_matched, family=binomial)

**Variable**	**aOR and 95% CI**	***P* value**
Non-beta-lactam SAP (Ref: Beta-lactam SAP)	1.68 (1.47-1.92)	<0.001

Abbreviations

aOR	Adjusted odds ratio
CI	Confidence Interval
SAP	Surgical Antimicrobial Prophylaxis
Ref:	Reference value

© 2025 Largiadèr S et al. *JAMA Network Open*.
