# Supplementary material for: β-Lactam vs Non–β-Lactam Antimicrobial Prophylaxis and Surgical Site Infection
Source: JAMA Netw Open. 2025 Oct 31;8(10):e2540809. doi: 10.1001/jamanetworkopen.2025.40809 (PMC12579348; doi:10.1001/jamanetworkopen.2025.40809)
Supplement: Supplement 2. — Nonauthor Members of the Swissnoso Group [file jamanetwopen-e2540809-s002.pdf]

Supplemental Online Content: Nonauthor Collaborators

*Indicates required information. Only first name, last name, and suffix will appear in PubMed.

*Group Name(s): Swissnoso							
***First Name and Middle Initial(s)**	***Last Name**	***Suffix (eg, Jr, III)**	Academic Degrees	Institution	Location (city, state/province, country)	Role or Contribution, eg, chair, principal investigator	Group (if more than 1 Group listed in the byline) and/or Subgroup (eg, Steering Committee)
Carlo	Balmelli		MD	EOC	Lugano, Switzerland	Swissnoso Member	
Niccolo	Buetti		MD	Geneva University Hospitals and Faculty of Medicine	Geneva, Switzerland	Swissnoso Member	
Philipp	Jent		MD	University of Bern	Bern, Switzerland	Swissnoso Member	
Hugo	Sax		MD	University of Zurich	Zurich, Switzerland	Swissnoso Member	
Matthias	Schlegel		MD	St. Gallen Cantonal Hospital	St. Gallen, Switzerland	Swissnoso Member	
Alexander	Schweiger		MD	Cantonal Hospital	Zug, Switzerland	Swissnoso Member	
Laurence	Senn		MD	University of Lausanne	Lausanne, Switzerland	Swissnoso Member	
Sarah	Tschudin-Sutter		MD	University of Basel	Basel, Switzerland	Swissnoso Member	
Danielle	Vuichard-Gysin		MD	Cantonal Hospital Thurgau	Münsterlingen, Switzerland	Swissnoso Member	
Aline	Wolfensberger		MD	University Hospital Zurich and University of Zurich	Zürich, Switzerland	Swissnoso Member	
Walter	Zingg		MD	University Hospital Zurich and University of Zurich	Zürich, Switzerland	Swissnoso Member	
