# Supplementary material for: β-Lactam vs Non–β-Lactam Antimicrobial Prophylaxis and Surgical Site Infection
Source: JAMA Netw Open. 2025 Oct 31;8(10):e2540809. doi: 10.1001/jamanetworkopen.2025.40809 (PMC12579348; doi:10.1001/jamanetworkopen.2025.40809)
Supplement: Supplement 3. — Data Sharing Statement [file jamanetwopen-e2540809-s003.pdf]

Data Sharing Statement

Largiadèr. β-Lactam vs Non–β-Lactam Antimicrobial Prophylaxis and Surgical Site Infection. *JAMA Netw Open*. Published October 31, 2025. doi:10.1001/jamanetworkopen.2025.40809

Data
Data available: No

Additional Information
Explanation for why data not available: Explanation for why data not available: Data are available upon reasonable request. For the process we refer to the Swissnoso data and publication regulations: https://swissnoso.ch/forschung-entwicklung/reglemente
